# Some challenges and opportunities for grazing dairy cows on temperate pastures

**DOI:** 10.1111/gfs.12458

**Published:** 2019-12-02

**Authors:** J. Michael Wilkinson, Michael R. F. Lee, M. Jordana Rivero, A. Thomas Chamberlain

**Affiliations:** ^1^ School of Biosciences University of Nottingham Loughborough UK; ^2^ Bristol Veterinary School University of Bristol Langford UK; ^3^ Rothamsted Research Okehampton UK; ^4^ Chalcombe Wickham UK

**Keywords:** grazed pasture, grazing management, herbage intake, milk production, milk quality, nitrogen use efficiency

## Abstract

Grazing plays an important role in milk production in most regions of the world. In this review, some challenges to the grazing cow are discussed together with opportunities for future improvement. We focus on daily feed intake, efficiency of pasture utilization, output of milk per head, environmental impact of grazing and the nutritional quality to humans of milk produced from dairy cows in contrasting production systems. Challenges are discussed in the context of a trend towards increased size of individual herds and include limited and variable levels of daily herbage consumption, lower levels of milk output per cow, excessive excretion of nitrogenous compounds and requirements for minimal periods of grazing regardless of production system. A major challenge is to engage more farmers in making appropriate adjustments to their grazing management. In relation to product quality, the main challenge is to demonstrate enhanced nutritional/processing benefits of milk from grazed cows. Opportunities include more accurate diet formulations, supplementation of grazed pasture to match macro‐ and micronutrient supply with animal requirement and plant breeding. The application of robotics and artificial intelligence to pasture management will assist in matching daily supply to animal requirement. Wider consumer recognition of the perceived enhanced nutritional value of milk from grazed cows, together with greater appreciation of the animal health, welfare and behavioural benefits of grazing should contribute to the future sustainability of demand for milk from dairy cows on pasture.

**Highlights**

- Grazing plays a central role in the nutrition of dairy cows in many regions of the world.
- Challenges to efficient management of grazed pastures include variable herbage supply and low herbage intake, which limit milk output per head.
- Opportunities for improved efficiency include more accurate diet formulation, breeding superior plant cultivars to improve ruminal efficiency and enhance product nutritional quality, application of robotics, sensors and artificial intelligence to improve grazing management and pasture utilization, and wider consumer recognition of the perceived enhanced value of milk from grazed cows.

## 1 INTRODUCTION

Herds of dairy cattle grazed on grasslands are ubiquitous worldwide, and their daily management ranges from semi‐nomadic to closely controlled intensive grazing (FAO, 2009). The emphasis in many regions is on production per head, especially where grazing plays a central role in the sustenance of the family and local community or where nutritional status of the individual animal is a key aesthetic attribute (Coote, 1992). On the other hand, in pasture‐based production systems in temperate regions, the capital cost of land is a relevant component of the cost structure of the system and is fixed. Therefore, production per hectare (ha) is often a more relevant factor than production per head (Macdonald et al., 2017). One of the main challenges is to balance stocking rate (SR), that is livestock units/ha, so that high levels of milk production per cow are achieved while maintaining high levels of pasture utilization per ha (Macdonald, Penno, Lancaster, & Roche, 2008). Therefore, the optimum SR is that which gives the maximum sustainable profitability per ha (Baudracco, Lopez‐Villalobos, Holmes, & Macdonald, 2010). Consequently, to improve production per cow or per ha, better control of herbage growth, through improved grazing management, and prediction of herbage growth, are critical challenges that pasture‐based farmers face.

With increased global demand for livestock products (FAO, 2009), the central role of grazed farm livestock in temperate regions in influencing total product output per head of livestock and milk and meat quality should be recognized. Increasing global competition for limited arable land, that can produce either energy crops, human food or animal feed, highlights the key role of grazing livestock in producing human food from less‐productive land that cannot (economically or physically constrained) be cultivated. This topic has been discussed elsewhere (Thornton, 2010; Van Zanten, Mollenhorst, Klootwijk, Middelaar, & Boer, 2016; Wilkinson & Lee, 2018), as has the contribution of grazing to the provision of ecosystem services (D’Ottavio et al., 2018) and these aspects are not considered here.

In the context of individual dairy herd size, there is a trend for increased herd size regardless of production system. Average herd size, annual milk yield per cow and annual output of milk per herd in 1986 and 2016 for four contrasting milk production systems are illustrated in Table 1, using national statistics for average herd size and milk yield per cow for New Zealand, the United Kingdom, Ireland and the United States. The emphasis on production per ha of grazed pasture in New Zealand has led to the development of large herds of intensively grazed spring‐calved dairy cattle, often on irrigated pasture to maintain forage production throughout the grazing season, with minimal inputs of silage and concentrate feeds. However, since the early 2000s, farmers have been increasingly seeking new feeds to supplement their pasture‐based systems, which has led to increased use of home‐grown or imported concentrate and forages (Clark, Caradus, Monaghan, Sharp, & Thorrold, 2007). In contrast, milk production in the United Kingdom is typically based on grazing in the summer months and conserved forage plus concentrates in the winter period. Irish dairy production systems are less intensive than the UK’s, that is, grass‐based with lower milk yields and smaller farms. In the United States, the emphasis is on total mixed rations (TMR) with significant reliance on cereal grain‐based concentrate feeds (Wilkinson & Lee, 2018).

**Table 1:** Average size of dairy herd, average annual milk yield per cow and average annual output of milk per herd in New Zealand, United Kingdom, United States and Ireland in 1986 and 2016 (AHDB, 2017; DEFRA, 2017; Donnellan, Hennessey, & Thorne, 2015; European Commission, 2018; LIC & DairyNZ, 2017; Milk Marketing Board, 1986; Pangborn, 2012; USDA, 2018)

	Herd size (cows/herd)		Milk yield (L/cow)		Herd output (‘000 L/herd)	
	1986	2016	1986	2016	1986	2016
New Zealand	145	414	3,062	4,259	444	1,763
United Kingdom	66	143	4,880	7,636	322	1,092
United States	89	327	7,553	10,328	672	3,377
Ireland	25	73	3,800	5,637	95	412

Changes in herd size (twofold to threefold increases) and milk yield (37%–57% increases) over the 30‐year period from 1986 to 2016 were broadly similar in all four countries. Herd size increased substantially, and it is notable that average herd size was higher in New Zealand than in other countries both in 1986 and in 2016. The increase in average herd size (269 cows/herd) was also greater in New Zealand than in other countries. However, average yield per cow was markedly lower in New Zealand than that in the United States (59% lower) and United Kingdom (37%–44% lower), and only 12%–18% lower than that of Ireland, reflecting the predominance of grazed pasture in milk production in New Zealand and Ireland. The relative increase in average annual milk yield per cow between 1986 and 2016 was similar for all four countries (37% for the United States, 39% for New Zealand, 48% for Ireland and 56% for the United Kingdom), with an average rate of increase of 1.5% per annum, reflecting genetic gain, improved nutrition, better animal health, higher fertility and advances in herd management. Despite a lower average herd size, output of milk per herd was 51% higher in the United States than in New Zealand in 1986 and almost double in 2016, reflecting the large difference in average annual milk output per cow between the two countries.

Soriano, Polan, and Miller (2001) concluded that pasture performed better than TMR regarding income over feed costs and, as cost of TMR decreased, the difference between income over feed costs between the different diets decreased. In contrast, Tozer, Bargo, and Muller (2003) reported that a TMR feeding system generated a higher net income than a grazing plus concentrate system. However, when feed and milk price were varied, that is, a combination of high feed prices and low milk prices, the grazing plus concentrate system was better than the TMR system in 17 out of 49 scenarios analysed. When milk prices are high, grazing systems may not be capable of making as much profit as TMR‐based production systems due to limitations in milk yield per cow. Level of pasture supplementation also has a significant effect on profit; increasing the amount of supplement, especially at lower milk prices, is reflected in decreased profitability (Macdonald et al., 2017; Patton, Shalloo, Pieerce, & Horan, 2012; Ramsbottom, Horan, Berry, & Roche, 2015).

The focus in this review is on some challenges and opportunities associated with the controlled grazing of temperate pastures by dairy cows in the context of feed intake, efficiency of pasture utilization, SR, output per animal, environmental impact and product nutritional quality, using examples from research investigating milk production from grazed pasture. Our hypothesis is that by recognizing the challenges and seizing new opportunities, producers of milk from grazed pasture can increase output, not by increasing herd size, but by increasing output per animal. Other important challenges such as adaptability of dairy cattle to current systems, improvement of longevity, productive lifespan and fitness (Knaus, 2009) and adaptation of the dairy grazing systems to climate change consequences (Gaughan, Sejian, Mader, & Dunshea, 2019; Pasqui & Giuseppe, 2019; Rust, 2019) are not addressed here.

Literature sources were used to provide evidence of the challenges to the grazing dairy cow in terms of daily voluntary dry‐matter (DM) intake (DMI), output per head and product quality. Examples were drawn from the literature of the extent to which grazed pasture may be complemented by other feeds in high‐yielding dairy systems. New opportunities for improved pasture management through plant breeding, robotics and artificial intelligence were identified from published and unpublished reports. Research on the influence of grazed pasture on milk composition, public perception and industry trends was reviewed to illustrate the potential to improve product composition and enhance consumer acceptability.

## 2 SOME CHALLENGES

### 2.1 Daily feed intake and pasture utilization

The main challenge to pasture management is to sustain, throughout the grazing season, a relatively constant daily supply of dense herbage comprising young vegetative growth of relatively high DM and energy density (McGilloway & Mayne, 1996). This must be achieved despite potentially large variations in forage growth rate due to season, temperature, water and nutrient supply. In addition, the feed intake of the dairy cow varies with live weight, milk yield and stage of lactation (Chamberlain & Wilkinson, 1996) and these factors, together with changes in herd size, must be balanced against forage supply to determine the daily area of land allocated to maintain target daily herbage allowance for the whole herd.

**Figure 1:**
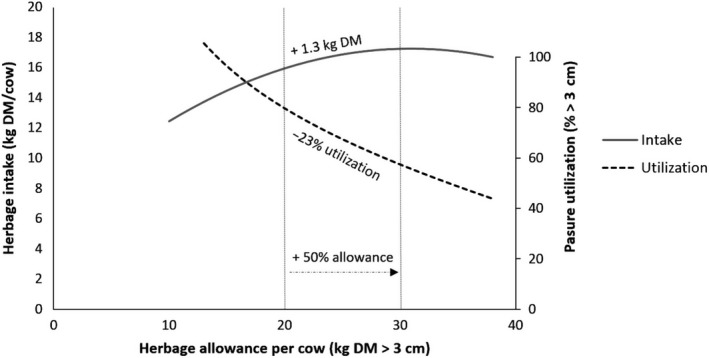
Relationships between herbage allowance, DM intake and efficiency of utilization of pasture (Derived from Baudracco et al., 2010)

Target levels of daily herbage allowance in intensive grazing systems are 20–30 kg DM/head (measured 3 cm above ground), to support a daily intake of 15–17 kg DM/head with an efficiency of utilization of pasture 3 cm above ground level between 50% and 70% (Figure 1).

**Figure 2:**
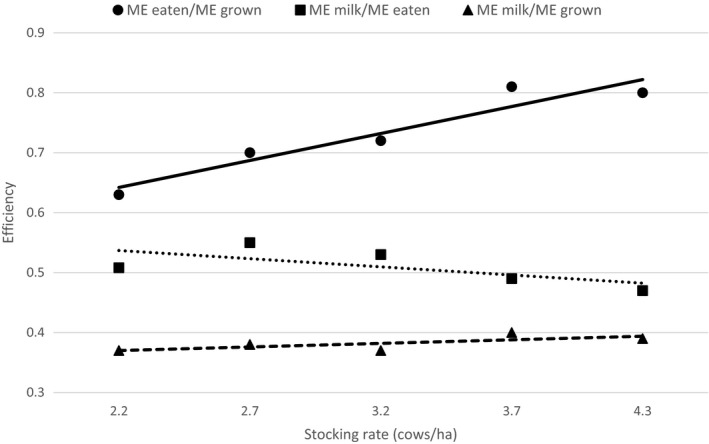
Effect of annual stocking rate on efficiency of metabolisable energy (ME) utilization (Macdonald et al., 2001)

Seasonal effects on efficiency of utilization of metabolizable energy (ME) within grazed pastures were investigated by Macdonald et al. (2001) who used a set of five farmlets to assess the effect on milk production of different annual herbage allowances, defined as kg live weight (LW) per tonne of herbage DM allowance per year. As comparative stocking rate was increased from 62 to 120 kg LW/t DM, equivalent to an annual SR increase from 2.2 to 4.3 cows/ha, ME intake increased as a proportion of ME grown (*p* = .016, Figure 2) while the proportion of ME converted to milk energy decreased (*p* < .001) due to reduced milk output per cow and a smaller proportion of consumed energy being allocated to milk production. However, there was no significant effect (*p* = .172) of SR on milk energy output per MJ of forage ME grown. On the other hand, McCarthy, Delaby, Pierce, Journot, and Horan (2011) studied the effect of varying SR from 1 to 5 cows/ha and found that an increase in SR of 1 cow/ha increased milk production/ha by 20% and reduced milk/cow between 7.4% and 8.7% compared with the production level of the lowest SR. These results indicate that there is no optimal SR to achieve the most efficient conversion of grass energy to milk energy at the animal level. However, McMeekan and Walshe (1963) consider that the optimum SR is such that the reduction in production/cow is 10%–12% of the potential production obtained at a low SR.

**Figure 3:**
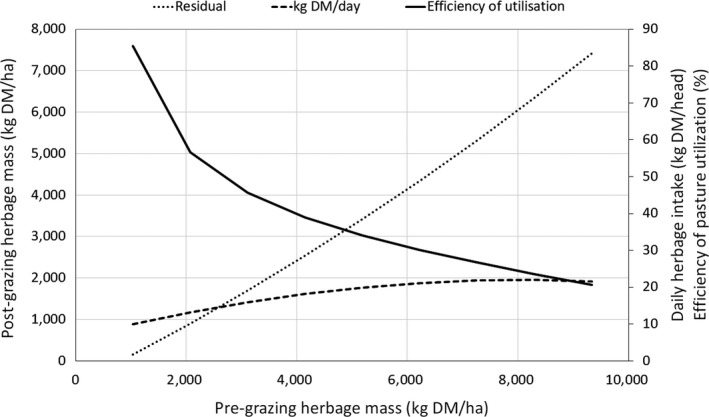
Relationship between pre‐grazing herbage mass per ha and post‐grazing herbage mass, herbage DM intake and efficiency of pasture utilization (Derived from Baudracco et al., 2010)

The effect of pre‐grazing herbage DM mass/ha (measured at ground level) on daily herbage intake/head reveals a quadratic relationship (Figure 3). As pre‐grazing herbage DM increases, the post‐grazing DM residual increases at a greater rate than that of the herbage DM consumption. Consequently, efficiency of pasture utilization declines from over 80% at 1,500 kg DM/ha to less than 20% at 9,500 kg DM/ha.

It is notable that the lines for daily herbage intake and residual (post‐grazing) herbage mass cross at about 2,700 kg DM/ha. This is close to the general advice to offer 2,600–3,000 kg DM/ha pre‐grazing and to graze to a post‐grazing residual of 1,500 kg DM/ha (DairyNZ, 2014). However, Pérez‐Prieto and Delagarde (2012, 2013) in their meta‐analysis concluded that the large range in dairy cow responses to variation in herbage allowance observed in the literature is largely explained by research methodology, that is, the height at which herbage mass and herbage allowance are measured (ground level, or at 2, 3, 4 or 5 cm above the ground).

**Figure 4:**
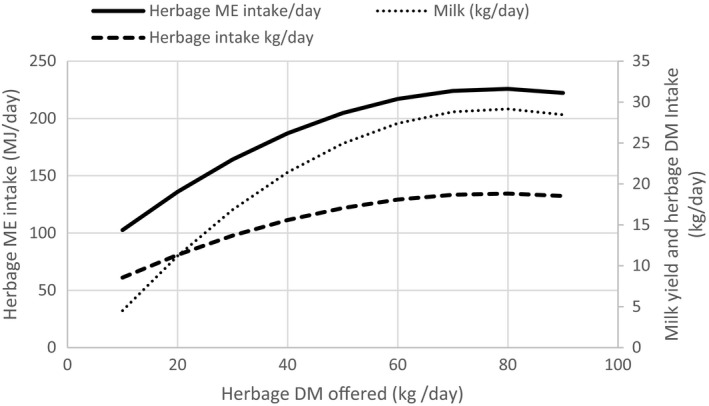
Relationships between daily herbage DM offered, herbage DM intake and milk output (Derived from Baudracco et al., 2010)

Relationships can be derived between daily herbage DM allowance, ME intake and milk ME output (Figure 4), assuming average grass energy value of 11.7 MJ ME/kg DM (Wilkinson, Allen, Tunnicliffe, Smith, & Garnsworthy, 2014), a daily ME requirement for a 650 kg dairy cow of 80 MJ ME/day for maintenance (Thomas, 2004) and milk energy content of 5 MJ/kg milk (AFRC, 1990). As the amount of herbage offered per cow increases, feed and energy intakes increase, reaching a maximum at a daily herbage allowance of approximately 70 kg DM/head (Figure 4). The effect of herbage allowance on milk yield is more pronounced at low herbage allowances, as increased energy intake dilutes the cow's maintenance requirement. However, as herbage DM allowance/head is increased, the amount of refused herbage also increases, leading to a decrease in herbage quality in subsequent grazing rotations (McEvoy et al., 2009). Increased SR, that is, decreased herbage DM allowance/animal, reduces post‐grazing herbage residual mass, resulting in a decrease in fibre (neutral detergent fibre (NDF) fraction) and increases in OM digestibility, ME and non‐structural carbohydrates (Macdonald et al., 2008) of the pasture. Therefore, the effect of herbage mass and daily herbage allowance on sward quality and milk production must be considered to achieve optimal production, both per cow and per ha (McEvoy et al., 2009).

### 2.2 Assessing herbage growth

Well‐managed grass has a high nutritive value and can meet feed requirements particularly in spring, summer and early autumn (Hennessy, Delaby, Pol‐van Dasselaar, & Shalloo, 2015). Offering high levels of high‐quality grass to spring‐calving cows results in high animal performance, although this can only be achieved with careful daily grazing management (O’Donovan, Ruelle, Coughlan, & Delaby, 2015). The long‐established standard method for assessing herbage mass is to cut samples of grass in small frames across the field area. Grass may be harvested to ground level or to a height comparable with the animal's grazing horizon (3–4 cm above ground level). However, this technique requires great effort and expense to collect enough samples to accurately represent a pasture, and farmers are not willing to make this effort in day‐to‐day management (Sanderson, Rotz, Fultz, & Rayburn, 2001). On the other hand, this method is used for training farmers on the visual assessment of herbage mass (O'Donovan, Connolly, Dillon, Rath, & Stakelum, 2002). Rising‐plate meter readings of sward density, calibrated against herbage mass (Earle & McGowan, 1979), are used on commercial farms to assess pre‐grazing and post‐grazing herbage DM mass/ha, but where there are large numbers of paddocks it is a time‐consuming activity with a high margin of error (Kallenbach, 2015). A major challenge is to engage more farmers in measuring grass growth rates and herbage mass on a regular basis, so that they can manage their pastures more precisely.

### 2.3 Output per cow

Cows must be fed appropriately to produce high yields, with due regard to breed and genetic merit. A high yield for a small Jersey cow might be typically around 4,000 L/lactation and containing 4%–5% fat and 3.5%–4% protein, whereas larger Holstein‐type cows can produce around 10,000 L/lactation containing less than 4% fat and about 3% protein (Dobson, Smith, Royal, Knight, & Sheldon, 2007). In addition to the intrinsic genetic potential for milk production and composition, feeding and management are major drivers of productivity/head.

Total mixed ration systems can support higher levels of output/head than pasture‐based systems. The main features of TMR are as follows: (a) no choice among feeds is permitted, (b) each bite is nutritionally balanced, (c) diets can be formulated for different cow categories (high producers, etc.), (d) DM and nutrient intakes are higher for TMR than for grazed pasture (Hofstetter, Frey, Gazzarin, Wyss, & Kunz, 2014; O’Neill et al., 2011; Schroeder, Couderc, Bargo, & Rearte, 2005), and (e) production of milk and total milk solids is higher for TMR diets than for grazed pasture (Gulati, Galvin, et al., 2018; Hofstetter et al., 2014; McAuliffe, Gilliland, Egan, & Hennessy, 2016; Schroeder et al., 2005; Vibart, Fellner, Burns, Huntington, & Green, 2008). However, milk solid concentrations are often lower for TMR than for forage‐based diets, and milk fat depression syndrome may occur under certain circumstances, although the syndrome can also be triggered in some grazing conditions (Rivero & Anrique, 2015). Nevertheless, the strongest argument in favour of grazed grass is the rising food versus feed debate with human‐edible foods (e.g. cereal grains) being used by ruminants given TMR diets (Wilkinson & Lee, 2018).

Challenges to output/head also include ketosis associated with rapid weight loss in early lactation, infertility, nitrate poisoning, hypomagnesaemia, diarrhoea, bloat and sub‐acute rumen acidosis (Wilkinson & Waldron, 2017). Some of these health issues are relevant in both pasture‐based and TMR‐based systems; however, the level of incidence varies greatly among farms under the same feeding regime (Shin et al., 2015). The decline in dairy cow fertility associated with higher genetic potential for milk production has had a negative impact on the profitability of dairy farms, especially those with seasonal calving pasture‐based systems (Shalloo, Cromie, & McHugh, 2014). Moreover, an extended calving interval, due to poor herd fertility, results in a breakdown in synchrony between pasture supply and demand, increased culling rates and replacement costs and reduced herd milk production, all of which reduce profitability (Shalloo et al., 2014).

The economic balance between milk output/cow and herd size is affected by the fixed cost structure of the farm. Furthermore, milk price and supplemental feed price play a relevant role on the system's profit through their direct effects on income and variable costs (Baudracco et al., 2010; Ramsbottom et al., 2015). The relationship between these prices is influenced by the considerable volatility of commodity markets (Macdonald et al., 2017). In the United Kingdom and United States, fixed costs/cow are high due to the considerable commitment to housing, feed storage, manure handling and associated labour. It is therefore desirable to maximize output/cow to achieve the best return on investment in fixed costs. By contrast, in New Zealand there is little housing, feed conservation or manure handling. The major fixed cost for New Zealand dairy units is that of land; output/ha is driven by increasing herd size and/or SR. Further, it seems illogical to maximize output/cow in the winter months and then in the summer to switch to a system that maximizes output/ha. The high fixed cost structure of housed systems usually demands that output/cow is maximized throughout the year. However, the best approach to follow may depend upon the relationship between milk price and supplemental feed price (Macdonald et al., 2017).

### 2.4 Efficiency of nitrogen use

The grazing animal has various effects on grassland, including treading and lying on herbage and distributing faeces and urine unevenly across the field, leading to nitrate and phosphate leaching, and nitrous oxide (N_2_O) emissions (Bilotta, Brazier, & Haygarth, 2007). The drive to increase output has been accompanied by higher levels of fertilizer application to grazed pastures and increased concentrate inputs to housed livestock units. A major environmental challenge is that these increased inputs, combined with increased SR, increase the risk of losses of nitrogen (N) and phosphorus (P) to soil, reflected in higher levels of phosphate and nitrate leaching and subsequent eutrophication of water courses. In New Zealand, for example, the increase in intensive milk production has been linked to significant increases in nitrate and dissolved P in more than 50% of the rivers monitored between 1998 and 2013 (Ministry for the Environment, 2015). However, this negative impact may be reduced in seasonal, spring‐calving, pasture‐based systems that import less than 5% of feed from off‐farm and that have no change in N fertilizer. Roche et al. (2016) observed that nitrate‐N (NO_3_‐N) leached/ha tended to decline with increasing SR in these dairy systems. This was associated with a reduction in days in milk/cow and a lower intake of crude protein (CP) during autumn, hence reducing N urinary excretion. Similarly, NO_3_‐N and total N concentrations in soil solution did not differ significantly between low, medium and high SR when N inputs from mineral N fertilizer or additional external feed supplements were only used when a feed deficit occurred and when increased grazed grass utilization was used to support the feed requirements of animals at higher SR (McCarthy et al., 2015).

Nitrogen excretion is directly related to N intake (Castillo, Krebreab, Beever, & France, 2000), and it has been estimated that at the relatively high concentration of CP in pre‐grazed herbage (typically in excess of 200 g/kg DM, Holmes et al., 2002; Wilkinson et al., 2014), average daily urinary N excretion by cows consuming 15 kg DM/day (Clement, Dalley, Chapman, Edwards, & Bryant, 2016) is about 250 g N/day (Wilkinson & Waldron, 2017). Although a variable proportion of excreted N is recycled into new forage growth, overall efficiency of N use by the grazing dairy cow is relatively low compared with indoor systems. Keim and Anrique (2011) reviewed this topic and reported that conversion of dietary N into milk N ranges between 13% and 31% in grazing systems and 40%–45% under confinement systems with balanced rations. At the whole‐farm level, N use efficiency (NUE) for different systems is highly variable (ranging between 8% and 64% of total N intake as milk N) with no clear superiority of one system over another (De Klein et al., 2017). Additionally, whole‐farm NUE is poorly related to whole‐farm N input (De Klein et al., 2017) and no clear relationship between whole‐farm NUE and milk production has been found (Gourley et al., 2012), indicating that farm management practices, soil type and climatic conditions are key drivers of whole‐farm NUE. Therefore, the challenge is to identify feasible strategies to increase efficiency of N use in the grazing situation.

### 2.5 Perception of milk quality by consumers

Production methodology is an important aspect of animal‐based products and milk is no exception. Milk processors and supermarkets are starting to favour grazing and grass‐based systems of milk production in response to consumer demand, linked to perceptions of enhanced animal welfare and milk nutritional quality. Strategies linking specific milk production systems with retailers rely on the consumer recognizing, seeking out and valuing such commitments which are often at a price premium and not simply selecting milk products based on fat content (“whole, semi‐skimmed and skimmed”) and price. The challenge is to demonstrate, for a range of grazing situations, not only improvement in animal welfare traits, but also enhanced product quality nutritionally (in terms of fatty acids, proteins and fat‐soluble vitamins) and for processing (dairy products and coffee milk foam).

## 3 SOME OPPORTUNITIES

### 3.1 Grazing management

Synchronizing herbage supply with demand is a fundamental objective of grassland management for dairy farmers on pasture‐based systems (Barrett, Laidlaw, & Mayne, 2005). The Teagasc Moorepark group recommends an early turnout of cows to pasture in spring with an average herbage mass of 600–700 kg DM/ha (measured at 4 cm above ground level), allocating between 10 and 13 kg DM per cow. For mid‐season, the recommendation is a pre‐grazing herbage mass of 1,300–1,600 kg DM/ha, while in autumn pre‐grazing herbage mass should be higher than in spring, with a target between 1,500 and 2,000 kg DM/ha (O’Donovan & Delaby, 2016; O’Donovan & McEvoy, 2016). But opportunities to make tactical adjustments to herbage supply can only be worthwhile if quantitative assessments of forage growth are made and, from that knowledge, likely levels of future growth are predicted. This approach has already been implemented in New Zealand where 5‐day forecasts of forage growth rate are produced for each local district from climate forecasts and current levels of soil moisture, calibrated to data for actual forage growth rate (DairyNZ, 2018).

Several systems have been developed that allow regular (i.e. weekly) pasture herbage mass estimates, either from a plate meter or from cutting and weighing/visual assessment, to be assimilated and used to guide pasture management. For instance, “PastureBase Ireland” is a web‐based grassland management application that helps farmers to determine the appropriate actions around pasture management (Hanrahan et al., 2017). INRA has also developed a spreadsheet that describes the evolution of the balance between grass growth and animal demand on a paddock‐by‐paddock basis (Delaby, Duboc, Cloet, & Martinot, 2015). Another Irish‐based system is commercially available for international use (Agrinet, 2018); registered users can define their farm and paddock set‐up and regularly enter plate meter readings. The data can be shared between groups and can be used to determine growth rates and forage availability “wedges” (i.e. a graph of paddock herbage mass for a dairy farm for a selected day, sorted by paddock from highest to lowest pasture herbage mass). Grazing groups can be defined to allow grazing allocations to be calculated and to assess productive performance of individual paddocks. Milk sales data and fertilizer applications can also be recorded to give a more detailed analysis of pasture performance. In the United Kingdom, plate meter readings and grass quality information are collected from 35 sites on a weekly basis by the Agriculture and Horticulture Development Board (AHDB) through their “Forage for Knowledge” scheme (AHDB, 2018). Growth rates, herbage mass and nutritional analysis are reported weekly and distributed freely through emails. Similar schemes such as GrassCheck GB supported by the Centre for Innovation Excellence in Livestock (CIEL), Rothamsted Research and AHDB have also recently been developed to follow the example set in Northern Ireland through the successful GrassCheck NI scheme (AFBI). Additionally, Teagasc in Ireland and INRA in France have developed dynamic models to predict the pasture growth considering weather conditions and N dynamics, for example the Moorepark‐St Gilles Grass Growth model. This is a user‐friendly model with basic and simple input which can react in a sensible manner to different weather and mineral N fertilization events (Ruelle, Hennessy, & Delaby, 2018).

Alternative devices to plate meter readings, using ultrasonic scanning, that can be towed behind an all‐terrain vehicle have been developed (Fricke & Wachendorf, 2013), but accuracy is lower in a moving sensor than for static assessments (Safari, Fricke, Reddersen, Mockel, & Wachendorf, 2016). In future, measurements of herbage mass could be automated using robotically controlled mini‐tractors (Yule, 2017) or by using electromagnetic reflectance at different wavelengths to develop vegetative indices to predict forage characteristics (Perez‐Sanz, Navarro, & Egea‐Cortines, 2017). However, more improvement would be required in these technological devices since results of an ultrasonic distance sensor assessed by Fricke, Richter, and Wachendorf (2011) were promising but could not exceed prediction accuracies ranging between *R* ^2^ = .80 and .82 in legume–grass mixtures (Fricke et al., 2011), and the accuracy decreases (from *R* ^2^ = .73 to .52) as the herbage mass increases (Safari et al., 2016).

Satellite imagery can be used to assess grassland herbage mass (British Grassland Society, 2017; Punalekar et al., 2018) but as cloud‐cover can interfere with some types of imaging, the information stream can be irregular. Where good images are obtained, there is a high correlation with herbage mass, but the correlation can be affected by atmospheric conditions such as high clouds and haze and therefore may not be consistent across recording dates. Other techniques such as Synthetic Aperture Radar (SAR; Moreira, 2013) are less weather dependent but at present are best at measuring herbage mass above 50 cm in height and are therefore not suitable for assessing grazing herbage mass. A terrestrial system using laser scanning and structure from motion (Cooper, Roy, Schaaf, & Paynter, 2017) successfully predicted above‐ground biomass of grass plots in the United States (*r* ^2^ = .54) and outperformed a standard plate meter (*r* ^2^ = .42). Images can also be collected using unmanned aerial vehicles (UAV) or drones; these machines are widely available but require a skilled operator to map paddocks and currently have limited flying time (20–30 min per flight). Where true normalized difference vegetation index (NDVI) images can be obtained, these can be combined with plate meter readings to predict pasture herbage mass (*r* ^2^ = .62–.77, *p* < .001; Andersson et al., 2017). As mentioned earlier, these technological methodologies would require improvement in the accuracy of prediction for the herbage mass range usually found in dairy grazing systems.

Hyperspectral imagery can also be used to predict herbage mass with acceptable accuracy (Geipel & Korsaeth, 2017). Images are collected at wavelengths ranging from 455 to 780 nm using specialist cameras mounted on UAV or small piloted aircraft, but the practicalities of regular data acquisition need to be resolved.

### 3.2 Future technologies to improve grazing

Opportunities to improve grazing efficiency include automated techniques for monitoring grazing behaviour and pasture allocation. For example, neck‐mounted accelerometers have been used to estimate grazing time (Oudshoorn et al., 2013) and are now being commercialized. However, data for grazing time and speed of biting derived from accelerometers are not good indicators of total daily herbage intake, because bite speed and bite mass vary with sward height, density and DM concentration (McGilloway & Mayne, 1996). Further work is needed to determine whether signals from neck‐mounted sensors can be used to predict intake when livestock are grazing pastures of varying composition and quality. Frequent allocation of daily herbage allowance may be beneficial to DMI and pasture utilization. Barrett, Laidlaw, Mayne, and Christie (2001) found an increase in daily DMI when pasture was allocated in three increments compared to a single daily allowance and Dalley, Roche, Moate, and Grainger (2001) showed a benefit to milk yield in early lactation from offering the daily pasture allocation of spring pasture in six portions compared to a single portion. The convenience in adopting this intensive management system would depend upon the benefits in productivity compared with potential increased labour costs.

“Frontal grazing” is the term used to describe an automated technique for incremental allocation of new areas of pasture to be grazed. The approach was launched commercially as the “Voyager” automatic grazing system (Lely, 2007) and was claimed to increase the efficiency of pasture utilization. The system comprised two solar‐powered wheeled robots with an electric fence strung between them. The robots could be programmed to advance slowly across a field, continually allocating new grass. The system was not widely adopted on commercial farms and is no longer available. Problems with varying terrain on commercial farms and the need to keep complex electronic devices separated from curious cows may have limited uptake. If such a system is to be developed further, thought needs to be given to the rate of allocation of new grass. Less frequent allocations would impair the full benefits of increased milk production. On the other hand, too frequent allocations would result in excess grass being allocated, high post‐grazing residuals and reduced pasture quality. It may be possible to use grazing behaviour, monitored with modern electronics, to determine when additional grass should be allocated. Developments since the 1990s with activity monitors for oestrous detection have produced robust hardware that can be integrated readily into commercial dairy herds (SCRDairy, 2018).

Virtual fences have scope for controlling grazing in livestock systems and have been reviewed by Umstatter (2011). Trials have been conducted with small groups of cattle given a variety of noxious stimuli to modify grazing behaviour (Bishop‐Hurley et al., 2007) and the “Boviguard” system has been developed in United Kingdom (Umstatter, Morgan‐Davies, & Waterhouse, 2015) based on GPS enabled neck collars on the cattle and induction cables buried in the pasture. The “Boviguard” system has been trialled in the Netherlands with good control of small groups of grazing animals (Hogewerf & Koene, 2018). Implementation of the system at a commercial level will involve substantial capital and structural investments, and these will need to be quantified against potential performance gains. A Norwegian review of the welfare aspects of the use of such electrical devices (Mejdell, Basic, & Bøe, 2017) revealed concerns regarding the unintentional and inappropriate delivery of shocks. The typical error of a standard GPS position fix is about 10m in any direction and it is likely that animals will work to smaller resolution than this when determining where they can and cannot graze. This could result in animals standing near the virtual boundary receiving warning stimuli at some times and not at others, which could hamper the learning experience.

Australian workers (Agersens, 2018) have recently brought to market a GPS‐based system of virtual pasture allocation developed and patented by CSIRO (CSIRO, 2006). The system consists of a solar‐powered GPS collar that tracks the animal's location relative to virtual boundaries defined as GPS co‐ordinates to create a virtual fence. Animals are trained to virtual fences by a combination of an auditory signal and a mild electrical pulse from the collar. The system could replace the need for permanent and temporary fences, especially long fencing runs in the Australian outback, and could also be effective in keeping animals away from environmentally sensitive areas such as river banks. There is evidence that in the period when the cows are learning the position of any virtual fence, they are not relaxed and graze less than when they are constrained by the visual cues of actual fences (Monod, Faure, Moiroux, & Rameau, 2009). The feasibility of transferring this technology from extensive systems to intensive grazing systems should be assessed.

### 3.3 Increasing dry matter intake

There is evidence that the low DM content in pasture can limit DMI (McGilloway & Mayne, 1996) and there may be an opportunity to increase intake through the selection of pasture species or varieties with higher DM or improved digestibility. The chemical composition of a forage determines the degradability of plant cell walls in the rumen, which subsequently influences the emptying of the rumen, which is decisive for DMI (Jung & Allen, 1995). Reducing the level of ferulate–polysaccharide–lignin complexes that cross‐link the cell wall has long been a target to enhance digestibility in grasses. Some natural variation has been exploited; for example, low xylan ferulate whole‐crop maize (*Zea mays* L.) gave improved milk yields (Jung, Mertens, & Phillips, 2011). This variation in cell wall composition could be pursued in pasture species such as perennial ryegrass (*Lolium perenne* L.) to increase digestibility, DMI and energy supply. Moreover, perennial ryegrass cultivars selected for increased water‐soluble carbohydrate (WSC) content have shown to increase the rate of fermentation (Purcell, Boland, & O’Kiely, 2014), which could increase DMI. Although significant effects of increased WSC of forage on DMI of sheep (Fraser, Fleming, Theobald, & Moorby, 2015) and steers (Lee et al., 2002) have been observed, this effect has not been seen in grazing dairy cows (Taweel et al., 2006). Nevertheless, even though Taweel et al. (2005) and Miller et al. (2001) did not find any effect of WSC content of zero‐grazed forage on DMI, the latter reported an increase in digestible DMI and milk yield driven by an increase in DM digestibility of forage selected for greater WSC content. This aligns with the findings of Moorby, Evans, Scollan, MacRae, and Theodorou (2006) who found increases in DMI and DM digestibility in cows with ad libitum access to varieties of zero‐grazed herbage either high or low in WSC, although no effect was observed on milk yield. Although genetic selection for improving DMI can be pursued, breeding new perennial ryegrass cultivars is a long‐term process (15–20 years) and gains would take time to become evident. Moreover, genotype × environment interactions can operate in some agroclimatic conditions (Parsons et al., 2011; Rivero et al., 2019) which might prevent cultivars from expressing the high‐WSC trait.

Managing sward composition may be another tool for improving herbage DMI and animal productivity by enhancing nutrient intake and utilization with lower inputs (Lüscher, Mueller‐Harvey, Soussana, Rees, & Peyraud, 2014). White clover (*Trifolium repens* L.) and red clover (*Trifolium pratense* L.) have high levels of CP (Tomić et al., 2014) and minerals, although relatively low concentrations of WSC compared with perennial ryegrass. Besides, organic matter digestibility, net energy concentration and the supply of metabolizable protein are generally higher for white clover than for grasses (Lüscher et al., 2014). Moreover, the authors reported that voluntary intake of legume forage can be 10%–15% higher than that of grasses of similar digestibility, and Harris, Auldist, Clark, and Jansen (1998) concluded that DMI was maximal when white clover was included at 60% in a forage mixture with grass. Egan, Lynch, and Hennessy (2017) observed that cows grazing a grass‐white clover mixture had greater average daily milk yield than cows grazing grass swards (18.6 vs. 17.0 kg/day), reflecting improved sward nutritional quality later in the season when the proportion of white clover was higher. Additionally, non‐grass species such as legumes and forbs have produced similar DM yields to a perennial ryegrass‐white clover mixture (Elgersma, Søegaard, & Jensen, 2013). Roca‐Fernández, Peyraud, Delaby, and Delagarde (2016) found that cows rotationally grazing a multispecies sward (grasses, clovers and forbs) ate 14.5% more DM, 12% more digestible OM, and produced 13%–21% more milk and 12%–15% more milk solids per day than cows grazing a single species sward of perennial ryegrass. These findings show that increasing sward botanical complexity under similar grazing management can have positive effects on animal performance (Roca‐Fernández et al., 2016).

The positive association between herbage allowance and intake may be exploited to increase daily herbage intake, but at the expense of efficiency of DM utilization. In general, a 50% increase in daily herbage allowance from 20 to 30 kg DM/head is associated with an increase in DMI of 1.3 kg/day and a 23% decrease in efficiency of utilization of available forage (Figure 1). Similarly, McDonagh, Gilliland, McEvoy, Delaby, and O’Donovan (2016) found that increasing herbage DM allowance from 15 to 20 kg/cow^−1^ per day^−1^ increases milk yield (11%) and solids (9%) but reduced sward utilization by 23%. The model shown in Figure 4 based on the experimental results from Baudracco et al. (2010) indicates that milk output would be maximal at 29 kg/day if cows were offered a daily herbage allowance of 80 kg DM/day (above ground level) and consumed 19 kg DM/day. More importantly, between 40 and 60 kg DM offered/cow, the response to the extra grass allowance was positive. The maximal grazed grass off‐take/ha in Figure 3 is at a pre‐grazing herbage mass of about 8,300 kg DM/ha and with a residual of 6,300 kg DM/ha (measured to ground level). However, the herbage offered at this high level of herbage mass would be tall material and the high level of residual pasture would include rejected plant stems, with reduced speed of forage regrowth and quality.

Supplementation of grazing animals with low‐cost by‐product feeds is a feasible option to increase total DMI. However, herbage DMI is substituted by supplement DM. The extent of this substitution rate plays a critical role on the efficiency of the feeding strategy, given its effect on the milk response to these supplements (Hills, Wales, Dunshea, Garcia, & Roche, 2015), since in general the greater the rate of substitution, the lower the average milk response to the supplement (Stockdale, 2000). Substitution rate depends on stage of lactation, type and amount of supplement offered, herbage mass and daily herbage allowance (de Klerk, 2012). A reasonable assumption would be a substitution rate of 0.7 kg decrease in grazed herbage DMI/kg supplement DM (Holmes & Roche, 2007). However, lower substitution rates were predicted by Delagarde et al. (2011) who obtained values from the “GrazeIn” model varying between zero at a low daily herbage allowance (10 kg DM/day > 5 cm) to 0.6 at a high daily herbage allowance (24 kg DM/day > 5 cm) with an average substitution rate of 0.3 kg decrease in grazed herbage DMI/kg supplement DMI. The main factor determining the economic benefit of the supplement is the relationship between milk price and supplemental feed price (Macdonald et al., 2017).

### 3.4 Output per cow

The main opportunities for increasing output per head at grazing are as follows: (a) careful pasture management to ensure adequate provision of high‐quality herbage and (b) strategic supplementation with low‐cost, high‐energy feeds. Provision of high‐quality pasture throughout the lactation can support relatively high levels of milk output/cow. Roche et al. (2013) recorded a whole‐lactation milk yield (4% fat‐corrected, FCM) of 6,651 kg in cows given a generous daily allowance of more than 45 kg DM cow^−1^ day^−1^, measured to ground level, indicating the potential of New Zealand Holstein/Friesian cows to produce milk from grazed pasture as the sole feed.

Supplementation of grazing dairy cows may be expected to increase milk yield, especially in early lactation. Responses by cows supplemented with a grain‐based low CP concentrate in the first 12 weeks of lactation are shown in Table 2. The response in milk yield during the first 12 weeks of lactation was 0.97 kg fat‐corrected milk (FCM)/kg supplement DM, similar to the mean response of 1 kg milk/kg concentrate DM in a meta‐analysis performed by Bargo, Muller, Kolver, and Delahoy (2003). Roche et al. (2013) found that milk protein percentage also increased with increasing level of concentrate supplementation but there was no effect of level of concentrate on body weight. As mentioned earlier, the economic value of this strategy would strongly depend on the price relationship between milk and supplemental feed. Importantly, Roche et al. (2013) recorded a significant increase in milk production in a three‐week period immediately after supplementation ceased (Table 2), though there was no residual effect of supplementation on milk composition. The residual response resulted in an overall response to supplementation in weeks 1–15 of lactation of 1.15 kg fat‐corrected milk (FCM)/kg concentrate DM. Total lactation yields for supplemented cows were 6,962 kg FCM for cows given 3 kg concentrate DM/day in the first 12 weeks of lactation and 7,231 kg FCM for cows given 6 kg concentrate DM/day. Conversely, Delaby, Faverdin, Michel, Disenhaus, and Peyraud (2009) found no carryover effect of the winter indoor feeding on milk yield of grazing cows after turnout, though there was a significant effect on milk composition, that is, fat and protein concentrations were higher under greater concentrate supplementation.

**Table 2:** Cumulative milk production (kg) over 43 wk from dairy cows grazing fresh pasture and offered 0, 3, or 6 kg (DM) of concentrates daily for the first 12 wk of lactation (Roche et al., 2013)

Weeks of lactation	Level of concentrate (kg DM/day)			SED	*p*
	0	3	6		
	4% fat‐corrected milk yield (kg/day)				
1–12	29.5	32.5	35.3	1.55	<.01
13–15	28.4	30.6	32.2	1.10	<.01
16–43	18.2	18.3	18.3	1.15	.99

Lipids, the most energy‐dense nutrients (38 MJ/g), are not fermented in the rumen and are directly available to the ruminant. As energy supply to ruminants on pastoral systems often limits output, a greater concentration of lipids in pasture grasses could significantly increase production for the same DM input. For example, an increase of 3% lipids in grasses could supply an extra 1.1 MJ of gross energy/kg DM, significantly increasing output, since 5 MJ metabolizable energy equates to 1 L of milk (AFRC, 1990). Forage lipids, typically unprotected in the rumen, account for only 3%–5% of total herbage DM, with the majority present in photosynthetic membranes of leaves. The energy content of the total diet may be increased by supplementing with oil to an upper limit of 60 g of unprotected oil/kg total diet DM, as higher levels have been shown to significantly reduce fibre degradability in the rumen (McAllan, Knight, & Sutton, 1983).

The total fatty acid concentration of a perennial ryegrass mapping family at the Institute of Biological, Environmental and Rural Science (IBERS), Aberystwyth, UK, showed a range from 1% to 5% of DM, and estimates of broad sense heritability of the main constituents ranged between 0.4 and 0.8 (Hegarty et al., 2013). These numbers are highly encouraging and indicate considerable scope for genetic improvement. Alternatively, approaches could be developed to increase the triacylglycerol (TAG) proportion of forage lipids, as with oil seeds. Triacyglycerol represents 0.1% of DM; however, several mutations that stimulate TAG synthesis and repress breakdown have been discovered that can substantially enhance TAG content in the leaves of the model plant *Arabidopsis thaliana* (L.) (Pant et al., 2015). The increase in fatty acid content is much larger than any so far achieved by conventional breeding and could be a basis for forage improvement programmes; but concerns regarding the impact on fibre digestion in the rumen with fatty acid supply greater than 6% of DM have first to be alleviated.

Recent research at Rothamsted Research in the United Kingdom (P. Eastmond, H. Van‐Erp, B. Vanselm, C. Hodgson and M.R.F. Lee, unpublished) compared *Arabidopsis* mutants with total lipids ranging between 7.2% and 8.1% of DM compared to wild type material with only 3.1%–4.0% lipids in DM, incubated for 12 hr in rumen fluid in vitro, to assess impact on DM and fibre digestibility. A third treatment comprised wild type + free oil to raise the total level of lipids to that of the mutant in the incubation (ca. 8%) was also included. Although there was no difference in fibre digestibility across the treatments, total NDF was lower in the mutant than the wild type 109 ± 25.2 versus 167 ± 16.9 g NDF/kg DM. Total DM disappearance during the 12 hr incubations was significantly reduced for the wild type + free oil than for the other treatments, with the mutant and wild type not different from each other. This suggests a lower impact on DM digestibility when the lipids were cellular bound rather than free oil, indicating potential for developing forages that express higher lipid content and do not reduce digestibility in the rumen. Animal‐based trials are required to validate this approach.

### 3.5 Efficiency of nitrogen use

Increasing resource‐use efficiency is associated with increased environmental sustainability. Grass‐based systems potentially are more efficient than indoor feeding systems because they use home‐grown feeds and minimize requirements for purchased feedstuffs and their associated resources of land, energy and machinery (Hennessy et al., 2015). Conventionally, diets based on conserved forages are supplemented with by‐products or compounded concentrates to supply sufficient energy and protein to meet animal requirements at an assumed level of total daily DMI (Chamberlain & Wilkinson, 1996). The content of CP in the total diet is about 180 g/kg DM for such diets. In view of the relatively high CP of grazed pasture herbage, noted above, the use of supplements of lower CP than that of grazed pasture would provide an opportunity to reduce total N intake and hence reduce urinary N excretion. For example, whole‐crop maize or whole‐crop wheat (*Triticum aestivum* L.) silage, included at 30% of total diet DM should give an overall level of CP of about 180 g/kg DM (Wilkinson & Waldron, 2017). However, as indicated previously, the consumption of supplement will reduce grazed herbage intake. Although herbage DMI is reduced, total DMI is increased and the reduction in total N intake is inversely related to substitution rate. Efficiency of supplementation depends on the relative costs of grazing and supplement, grazed herbage allowance, season and stage of lactation (Tozer, Bargo, & Muller, 2004).

In the study of Roche et al. (2013; Table 2), the higher level of concentrate supplementation reduced diet CP from 220 to 190 g/kg DM. Estimated NUE in the first 12 weeks of lactation was 27% for cows given no supplement and 36% for cows given 6 kg concentrate DM/day, indicating an opportunity to increase NUE through concentrate supplementation in early lactation. This improvement in NUE should be evaluated at the system level to assess the impact of the supplementation strategy on the system's environmental impact. In this regard, a great variation between farms and between catchments has been reported by De Klein et al. (2017) for some N‐related indicators. The authors also highlight that realistic goals for both NUE and N surplus depend on the agroclimatic context of the dairy system and on its economic and environmental goals.

An alternative to reduce the CP content of herbage could be to add forbs to the pasture mixture; Totty, Greenwood, Bryant, and Edwards (2013) found that a diverse pasture mixture (high‐sugar perennial ryegrass + white clover + chicory (*Cichorium intybus* L.) + plantain (*Plantago lanceolata* L.) + lotus (*Lotus pedunculatus* L.)) had lower CP, more WSC, and less fibre than a high‐WSC grass/white clover mixture. This difference in nutritional composition led the diverse sward to favour all N metabolism indicators (plasma urea, urine N, urea N, urine NH_3_, N output, milk urea N, and milk N excretion) and improved milk yield when compared with cows grazing the grass/legume swards.

Higher‐WSC grasses potentially offer a better balance between rumen‐degradable protein and fermentable carbohydrates in pasture, resulting in greater efficiency of N use (Lee et al., 2003; Miller et al., 2001) although this potential is not always realized (Tas et al., 2006a, 2006b). Alternatively, supplementation with fermentable carbohydrates, for example, grain or sugar beet (*Beta vulgaris*) pulp or “soft” NDF grass varieties is an effective strategy to increase microbial capture of excess protein (Sinclair, Garnsworthy, Mann, & Sinclair, 2014). Other improvements centre around either reduction in total protein supply or reduction in availability of forage N in the rumen. In a review by Abberton and Marshall (2005), the authors indicate the importance of breeding goals for forage legumes to reduce their contribution to both direct (leaching) and indirect (through animal returns) N and P pollution. This would predicate the breeding of forage legumes such as white clover with lower levels of total N and properties associated with mechanisms affecting protein breakdown in the rumen and silo. One such target has been identified as polyphenol oxidase (PPO) in red clover (*Trifolium pratense* L.) which has been shown to improve NUE through reducing the digestibility of plant protein in the rumen and increasing the proportion of dietary protein flow out of the rumen (Lee, 2014).

### 3.6 Enhancing milk quality

For animal‐based food products, aspects of quality relate to taste (flavour hedonics), health (human well‐being) and production method (“naturalness”). Differences in taste between milk and cheese from grass‐based and other production systems have been reviewed by Kilcawley, Faulker, Clarke, O’Sullivan, and Kerry (2018). They concluded that untrained assessors, who best represent consumers, were less able to discriminate sensory differences than trained panellists and that visual and texture attributes, primarily driven by changes in fat, protein and β‐carotene content, were more likely to be identified than any flavour attributes.

Grazed pasture, as opposed to indoor feeding based on silage and concentrates, generally leads to increased levels of beneficial milk fats, for example, polyunsaturated fatty acids (PUFA), especially omega‐3 PUFA and conjugated linoleic acid (CLA; Butler et al., 2009; Dewhurst, Shingfield, Lee, & Scollan, 2006; Elgersma, 2015; O’Callaghan et al., 2016). Surveys have also shown that milk fat profiles are significantly altered between summer milk produced from cows grazing fresh pastures and winter milk from conserved forages (Agenäs, Holtenius, Griinari, & Burstedt, 2002; Jahreis, Fritsche, & Steinhart, 1997). Increasing total lipids in grasses can also improve the supply of omega‐3 PUFA to the animal, which has beneficial effects in terms of animal health and fertility (Richardson, McNiven, Petit, & Duynisveld, 2013). Concerns have been raised about the potentially negative effects on human health of animal fat through over‐consumption (Garnett, 2009). However, forage‐fed animals produce meat and milk with a lipid composition more favourable to human health than that derived from concentrate‐fed animals (Daley, Abbott, Doyle, Nader, & Larson, 2010). This is particularly associated with a more beneficial ratio of omega‐3 to omega‐6 PUFA as forages have a high omega‐3 PUFA content with total lipids typically comprising 50%–75% 18:3n‐3 and 6%–20% 18:2n‐6 (Dewhurst et al., 2003). Pasture species with higher fatty acid content, specific enzymes, for example PPO (Lee, 2014), plant secondary metabolites (Buccioni, Decandia, Minieri, Molle, & Cabiddu, 2012) or green odour compounds (Huws, Scott, Tweed, & Lee, 2013), that improve capture of PUFA across the rumen and enhance the beneficial impact of forage feeding would be an important tool in meeting the needs of the value chain and consumers.

As well as the impact on milk fat of pasture grazing compared to indoor feeding, O’Callaghan et al. (2016) reported higher concentrations of total protein and casein in milk from grazed cows compared to milk from cows given diets based on silage and concentrate. Furthermore, Manzi and Durazzo (2017) reported higher levels of fat‐soluble vitamins (β‐carotene and α‐tocopherol) in pasture‐based organic milk than conventional milk, which could further improve the “health” quality of the milk. However, the study reported that the differences, in terms of the European Food Safety Authority (EFSA) Population Reference Intakes (EFSA, 2017), were negligible at the level of standard milk servings. Manzi and Durazzo (2017) also indicated a significant reduction in iodine content of pasture‐based organic milk compared to conventionally produced milk where cows are given mineral mixtures and concentrates containing supplementary iodine and selenium (Se), which is involved in iodine metabolism. Pastures are often found to contain relatively low levels of iodine and Se and, in organic systems with more diverse swards, the grazing animal may be more at risk to potential iodine antagonists such as glucosinolates.

Milk and milk products produced on pasture‐based systems can therefore be considered as a functional food, exhibiting health benefits beyond their nutritional value. In addition to being a source of proteins, lipids, vitamins and minerals, milk exerts other beneficial properties due to the presence of numerous bioactive molecules (Descalzo et al., 2012). Milk derived from pasture‐based diets contains higher levels of natural antioxidants, a particular profile of volatile compounds and a higher content of functional fatty acids.

There is evidence of growing consumer preference for milk from grazed livestock. For example, supermarkets and milk buyers in Europe are setting targets and standards that require dairy cows to graze pasture during the summer, that is, cows grazed at pasture for at least 120 day per year and at least 6 hr/day. This initiative has resulted in an increase in the percentage of dairy farms grazing (Van den Pol‐van Dasselaar, Becker, Botana Fernandez, Hennessy, & Peratoner, 2018). In the United Kingdom, the supermarket chain Marks and Spencer Ltd now requires that milking cows are provided with access to grazing for at least 100 days for at least 4 hr/day and farmers are encouraged to provide pasture access whenever weather conditions permit as part of their “Plan A 2025 Sustainability Plan” (Marks & Spencer, 2018). Similarly, Waitrose Partners Ltd requires that most of the cows in a herd graze for at least 4 hr a day for a minimum of 120 days a year (Waitrose, 2019).

Of course, access to grass does not ensure grazed herbage intake. Other UK groups have gone further, with the producer group “Free‐Range Dairy Farmers,” supplying the supermarket chain Associated Dairies Ltd (ASDA), contracting producers who sign up to the “brand” to 180 days and nights a year at pasture as part of their “Pasture Promise” (Free Range Dairy Farmers, 2017). Other countries have taken a policy view to grazing with the Swedish Animal Welfare Ordinance requiring that cattle are grazed outside at pasture in the summer (Government Offices of Sweden, 2009).

The added value of grazing production systems is clear to consumers who wish to purchase a more naturally produced product. There is also anecdotal evidence that milk from local pasture‐based farms is preferred in coffee houses due to the quality of the milk foam produced, possibly due to the difference in protein and fat content in pasture‐based milk compared to other sources of milk (Gulati, Hennessy, et al., 2018). This feature requires further investigation and could be a huge opportunity in adding value to the production of pasture‐based milk, due to the current global increase in coffee consumption (Hicks & Halvorsen, 2019).

Assessments of negative animal welfare such as lameness and mortality are recorded routinely on many larger‐scale dairy farms; it is increasingly important to record positive assessments of animal welfare based on behavioural motivation (Mellor, 2015). Such approaches will capture the increased ability to express natural behaviour at grazing and will further add value to milk produced from grazed pasture.

## 4 CONCLUSIONS

Grazing presents a range of challenges including variable and unpredictable herbage growth, lower daily herbage intake, lower output per animal, lower efficiency of N use, and inefficient grazing management. Opportunities exist to develop novel approaches to grazing management to meet these challenges, including the application of automated techniques for monitoring grazing behaviour and pasture allocation, based on remote sensing and hyperspectral imaging, to integrate animal behaviour with pasture allowance. Developments in plant breeding also provide potential routes to improve animal performance by aligning animal requirements with forage nutritional content. Advantages to milk quality (health and naturalness) attributed to pasture‐based systems are recognized by discerning consumers and are being exploited by producer groups and supermarket chains.
